# A Bionic Spatial Cognition Model and Method for Robots Based on the Hippocampus Mechanism

**DOI:** 10.3389/fnbot.2021.769829

**Published:** 2022-01-14

**Authors:** Jinsheng Yuan, Wei Guo, Fusheng Zha, Pengfei Wang, Mantian Li, Lining Sun

**Affiliations:** ^1^State Key Laboratory of Robotics and System, Harbin Institute of Technology (HIT), Harbin, China; ^2^Shenzhen Academy of Aerospace Technology, Shenzhen, China

**Keywords:** hippocampus, intelligent robot, bionic cognition, grid cells, robot perception

## Abstract

The hippocampus and its accessory are the main areas for spatial cognition. It can integrate paths and form environmental cognition based on motion information and then realize positioning and navigation. Learning from the hippocampus mechanism is a crucial way forward for research in robot perception, so it is crucial to building a calculation method that conforms to the biological principle. In addition, it should be easy to implement on a robot. This paper proposes a bionic cognition model and method for mobile robots, which can realize precise path integration and cognition of space. Our research can provide the basis for the cognition of the environment and autonomous navigation for bionic robots.

## Introduction

Biological intelligence processes information using neuronal discharge, which makes those living creatures able to perceive and act in the real world exceptionally well. Imitating neural systems to realize robot navigation has attracted the interest of many researchers (Bing et al., 2018, 2019). The hippocampus and its accessory in the brain are the core physiological regions for environmental cognition and navigation (Eichenbaum, 2017; Gu et al., 2018; Steven et al., 2018). As early as O'Keefe and Dostrovsky (1971) found some nerve cells in the hippocampus of rats with specific expressions for places. These cells would fire when the rat was at a specific place, and were named “place cells” (O'Keefe, 1976). In Hafting et al. (2005) found there were nerve cells with strong periodic firing characteristics in the entorhinal cortex. The firing field presented a hexagonal grid and covered the whole movement space. And then these nerve cells were named “grid cells.” In O'Keefe and Burgess (1996) found that when changing the space range, the firing field of grid cells would move toward the edge of the environment. So they forecast the existence of “boundary cells” with a firing reaction on the boundary, which could perceive the distance toward the environment boundary. By 2008, The researchers found boundary cells in the shallow of the olfactory cortex (Savelli et al., 2008). In 2012, O'Keefe and Burgess published research (Krupic et al., 2012) showing that cell clusters with periodic striped firing fields were found in the shallow layer of the paragentum and entorhinal cortex. These cell clusters had different firing orientations and wavelengths, and they were named “stripe cells.”

The anatomical experiments have found strong interconnections between various hippocampus regions, but the specific mechanism for information processing is still poorly understood. Studies show that cognitive maps exist in the brains of rats. During nesting, foraging, and other behaviors, the hippocampus can integrate movement paths and walk along the direction of the target through the path not experienced. How the hippocampus accomplishes these calculations is still inconclusive (Epstein et al., 2017; Sarel et al., 2017; Savelli and Knierim, 2019). Current studies in the field of neurobiology focus on the information processing mechanism from grid cell to place cell, but do not consider coupling velocity information to grid cells (Rolls et al., 2006; Si and Treves, 2009; Savelli and Knierim, 2010; Danjo et al., 2018). Burak et al. (2008) proposed a grid cell model based on the continuous attractor network (CAN), which can calculate the path integral accurately. However, their research is still limited in practical application: Firstly, the grid cells model based on a continuous attractor network can only explain the problem of path integration, but lacks the explanation of perceptual input and the cognitive output, so cannot build a cognitive map; Secondly, the method of realizing spatial cognition has the problem of accumulating errors, which can only keep the accuracy in a short time or a small space.

The University of Queensland, Australia, proposed a real-time localization and mapping method “RatSLAM” based on the spatial cognition mechanism of the rat's hippocampus. Inspired by place cells, they fabricated a “pose cells” attractor model, which uses velocity and direction information to drive the activity packets of “pose cells” on the neural plate, thus achieving path integration and expression (Milford and Wyeth, 2008). However, they mainly imitate the spatial cognitive methods of rats at the neurobehavioral level. They do not imitate the cognitive mechanism at the neurophysiological and anatomical levels. Besides, the hypothesis about “pose cells” does not conform to physiological facts, so this method has limitations in expansion and application.

Inspired by the biological mechanism of spatial cognition in the hippocampus mechanism of rats, this paper proposes a spatial cognition model and method that can be used in robots. The method conforms to the biological mechanism, and can achieve precise path integration and spatial cognition for a long time. This research will promote the development of research on bionic intelligent robot environment cognition and autonomous navigation systems.

## Models and Methods

In this section, based on the physiology and anatomy of spatial cells, we built five kinds of hippocampal cell models: head direction cells, stripe cells, grid cells, place cells, and boundary cells. We built a complete robot spatial cognition model based on these cell modes by combining encoding and decoding methods. Our method used the velocity and direction of the robot movement as the input information. Then the head direction cells coded this information as nerve signals. Then the head direction cells projected signals into the stripe cells. Then the cognition model obtained a one-dimensional path integral in the stripe cell. Then the stripe cells sent the one-dimensional signal to the grid cells. Finally, the cognition mode realized path integration and expression of place in grid cells. The place cells obtained the single-peak firing expression of the current position by decoding the multi-scale grid cells signal.

### Establishment of Spatial Cell Model

#### Head Direction Cells Model

**Figure 1:**
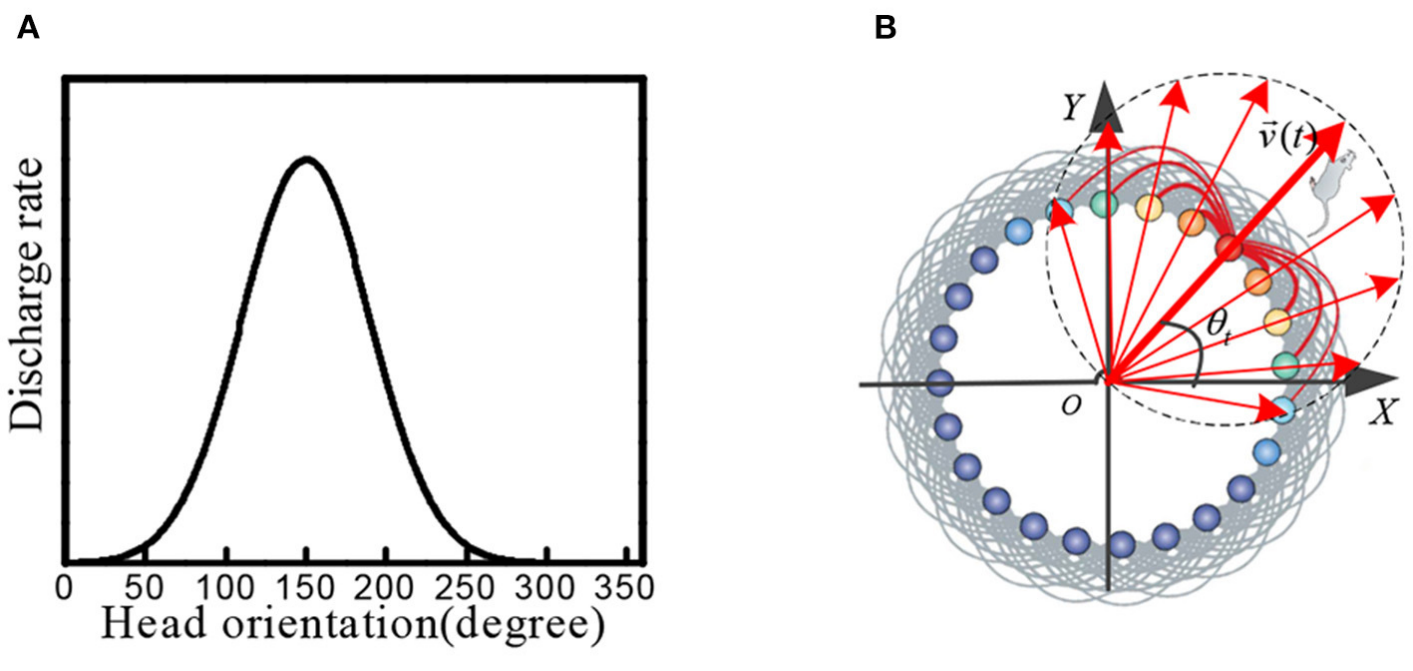
**(A)** Physiological firing characteristics of head direction cells. **(B)** In the attractor model of head direction cells, velocity and direction information is encoded into neural firing characteristics.

Head direction cells were identified in the posterior subiculum region of the hippocampus. These cells maximized firing when the animal's head was facing in a specific direction (Taube et al., 1990; Taube, 1995; Bing et al., 2021). When the head of the rat was facing a specific preferred direction, the maximum firing occurred. The firing gradually decreased when the head was away from this direction, as shown in Figure 1A.

We constructed a circular attractor model as the head direction cells, which encoded the direction and velocity information. This information was used as the input signal of stripe cells. As shown in Figure 1B, the head direction cells are arranged in a ring, and the position of each cell in the ring corresponds to the preferred orientation of the cell itself. The phase direction of the attractor activity lump represents the dominant orientation, which codes the direction of the rat's head. We established the outer Cartesian coordinate system with the center of the circular attractor as the origin. The head orientation at time *t* was set as θ_*t*_, and the movement velocity was set as *v*_*t*_, the phase angle of the *i*th head direction cell in the attractor model was set as θ_*i*_. The movement velocity was proportional to the firing rate of the head direction cell. Each head direction cell generated the firing rate signal *s*_*i*_(*t*), which contained the information of the current head at the angle and movement velocity:


(1) $$\begin{array}{l} s_{i}\left(t\right)=v\left(t\right)\cdot \cos\left(\theta_{i}-\theta_{t}\right) \end{array}$$

#### Stripe Cells Model

**Figure 2:**
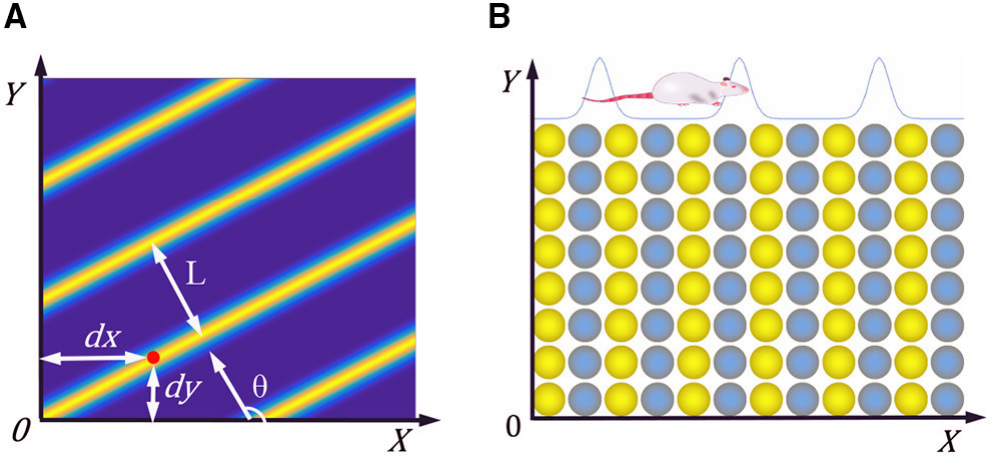
**(A)** Firing characteristic parameters of stripe cells. **(B)** Neural plate of stripe cells, neighboring neurons have synaptic connections in the *X*-axis direction, but not in the *Y*-axis direction.

The researchers found a kind of cell with periodic striped firing fields in the parietal underdrum and the superficial dermis of the entorhinal cortex in rats (Krupic et al., 2012). The characteristic firing parameters of stripe cells is shown in Figure 2A, θ represents the movement direction of the stripe, *L* represents the period distance of the stripe, and (*dx, dy*) represents the phase of the stripe cells. When the rat moves in a particular direction, the velocity in that direction is integrated and encoded.

We constructed the stripe cell neural plate based on the continuous attractor network, as shown in Figure 2B. There are synaptic connections between neurons in the length direction but no connection in the width direction. Each neuron can fire in response to movement in a specific direction, depending on the preferred direction of head direction cells. The velocity information received from the head direction cells drives the neurons' periodic firing, making a flowing striped wave on the neural plate. The position of the rat can be encoded according to the phase change of the striped wave.

In order to obtain striped firing characteristics, the weight of the synaptic connection between neurons in the length direction was set to mutual inhibition, and the velocity regulation signal generated by the head direction cells was used as the forward input of the stripe cells. Referencing the modeling method of continuous attractor network (Burak et al., 2008), the firing dynamics model of stripe cells could be constructed as follows:


(2) $$\begin{array}{l} \tau ds_{i}/d_{t}+s_{i}=f\left[\underset{j}{\sum }W_{ij}s_{j}+B_{i}\right] \end{array}$$

Where τ is the time constant of the neuron firing, and the neuron transfer function *f* is a nonlinear rectifier function by *f*(*x*) = *x*, for *x* > 0, and is 0 otherwise. The firing state of all the neurons in the current position is *s*_*i*_, and *W*_*ij*_ is the connection weight of the neuron from *j* to *i* in the stripe cell neural plate. $\underset{j}{\sum }W_{ij}s_{j}$ is the inhibitory input projected from neighboring neurons, and *B*_*i*_ is the forward excitatory input from the upstream head direction cells.

The connection's weight matrix of stripe cells is as follows:


(3) $$\begin{array}{l} W_{ij}=W_{0}\left({\vec{x}}_{i}-{\vec{x}}_{j}-k\cdot {\vec{e}}_{\theta_{j}}\right) \end{array}$$

**Figure 3:**
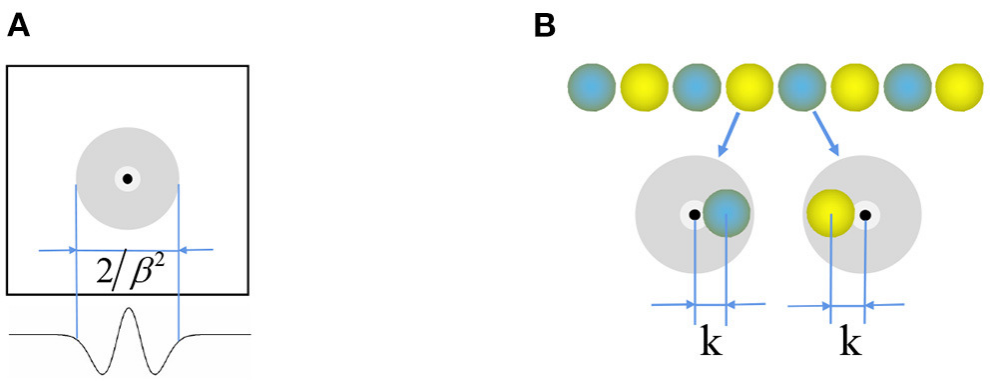
**(A)** Mexican hat weight profile for connection between the neurons. **(B)** Weight offset contour of stripe cells.

Where function $W_{0}\left(\vec{x}\right)=e^{-\gamma |\vec{x}{|}^{2}}-e^{-\beta |\vec{x}{|}^{2}}$, and ${\vec{e}}_{\theta_{j}}$ is the unit vector along the preferred direction θ_*j*_ of the neuron *j*. Figure 3A shows that the weight matrix forms a hat shape distribution with high in the middle and low on both sides. Each neuron in the stripe cell neural plate has a priority direction, the same as the priority direction of the upstream head direction cells. Furthermore, the priority direction of the left and right adjacent neurons *i* and *j* is opposite. The inhibitory weight matrix of the neuron to the surrounding neurons will shift toward its priority direction. According to the principle of “Turing pattern dynamics,” when there is only inhibitory input between neurons, the interaction between neurons causes the neural plate to form steady striped firing spontaneously. Half of the neurons are excited when there is a certain direction of the velocity input, and another half do not respond. So the original static striped firing balance is broken. The firing pattern of the stripe cells spontaneously shifts along the velocity direction. As shown in Figure 3B, the central position of the weight matrix of stripe cells is *x* − *k* and *x* + *k*. We set γ = 1.035 × β and β = 3/λ^2^, λ is the firing period on the neural plate. Since the *W*_0_ is always less than or equal to zero, all the connections are inhibitory, and locally surrounding inhibitory connections interact to generate a striped firing.

The forward input to the neuron is:


(4) $$\begin{array}{l} B_{i}=1+\alpha {\vec{e}}_{\theta_{i}}\cdot \vec{v} \end{array}$$

Where ${\vec{e}}_{\theta_{i}}$is the unit vector along the preferred direction of the neuron, and $\vec{v}$ is the unit vector in the direction of the current velocity of the rat. If the coefficient *k* or α is 0, static fringes are generated. If *k* and α are both nonzero, then the rat's velocity is coupled with the firing pattern of the stripe cell plate to drive the formation of a flowing striated wave. The *k* and α product determines the intensity of the streaks driven by the velocity input. The striated wave can only maintain a stable fringe pattern when the output weight offset *k* is relatively tiny. Based on constant *k*, the gain of the stripe cell network to the velocity response is determined by α.

#### Grid Cells Model

Researchers found grid cells in the second layer of the olfactory cortex in the rat hippocampus, but unlike the place and head direction cells, the grid cells were scattered and fired weakly. Grid cells in different regions along the dorsal and ventral axis in the entorhinal cortex have different grid periodic scales. The firing pattern of grid cells population in each scale is the same. The firing fields form a stable hexagonal pattern. The firing activity of grid cells does not depend on external cues, even if the rat's activity pattern does not change in a dark environment.

**Figure 4:**
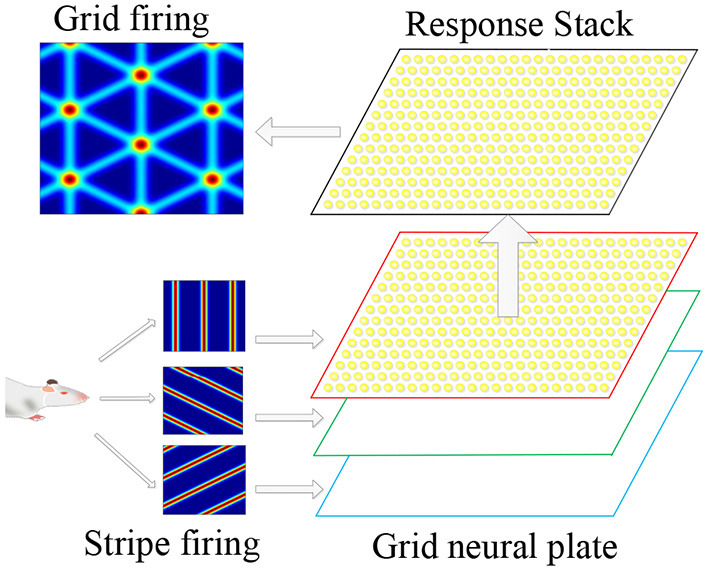
Schematic diagram of grid cells model coding principle. According to the movement information of the robot, the striped firing response is formed in the stripe cells. The striped firing signals are projected onto the grid cells, forming a grid-like firing response.

The superimposition of multiple stripe cell firings could form periodic grid cell firing fields, so the stripe cells were considered the primary mechanism for firing grid cells. Therefore, we proposed to use the stripe cells firing as the forward signal input of the grid cells, and multiple flowing striated waves jointly drove the grid cells to encode the space to form a flowing two-dimensional firing grid. This information transmission and processing method conformed to the physiological basis. As shown in Figure 4, it is a schematic diagram for the coding principle of the grid cells model. We used a two-dimensional continuous attractor model to model the grid cell population. Each attractor represents a grid cell, and its activity state is related to the forward input of the striped cells. We composed grid cells with the same firing cycle to form a neural plate, and projected the firing information of striped cells in different directions onto the neural plate, and generated superimposed firing responses on the grid cells, thus forming a grid firing. The velocity of the robot's motion drives the firing pattern of the striped cells to flow, and the grid cells also generate a flowing firing grid. So the grid cells model realizes the integration of the path information.

#### Place Cells Model

**Figure 5:**
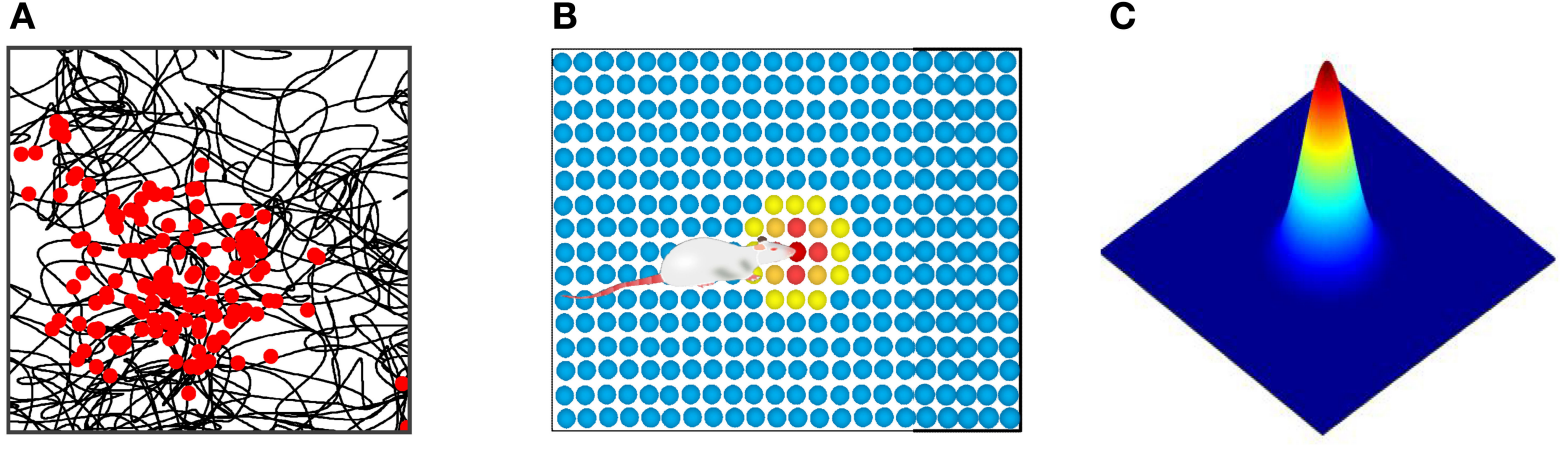
**(A)** Physiological firing characteristics of one place cell. The black curve represents the rat's trajectory, and the red dot represents the cell's firing at that location. **(B)** Neural plate model of place cells population. **(C)** The mathematical model of place cell's response to spatial location.

Place cells are a kind of spatial place-selective firing cell. For each place cell, only when the rat is in a specific position in space, the cell will make firing activity, but in other places in space it does not produce firing activity. Firing characteristics were found by physiological research as shown in Figure 5A, the black curve represents the trajectories of the rat, the red dot represents the firing position of place cells, place cells establish a one-to-one correspondence between neurons in the brain region and the physical world. We built, as shown in Figure 5B, a two-dimensional neural plate, the firing of the place cells population showed a single peak pattern, which reflected the spatial place of the robot. The place cells mathematical model proposed by O'Keefe et al. was adopted to calculate the firing rate of cells at various locations (O'Keefe and Burgess, 2010). Figure 5C shows the model of a single place cell's response to spatial location. Its mathematical expression is:


(5) $$\begin{array}{l} R_{pc}^{i}\left(r\right)=exp\left(-||r-r_{i0}|{|}^{2}/\delta^{2}\right) \end{array}$$

Where, $R_{pc}^{i}\left(r\right)$ is the firing rate of place cell *i* at position *r*, *r* = (*x, y*) representing the current position of rat in the environment. *r*_*i*0_ is the position corresponding to the firing field center of the place cell *i*, δ^2^ is the adjustment coefficient of the firing field of the place cells.

#### Boundary Cells Model

The recursive attributes of the path integral by grid cells cause the accumulated error to increase continuously. If there is no error correction mechanism, it will seriously damage the positioning accuracy. Researchers have long-term tracking experiments on rats in open spaces. They found that the accumulation error of grid positioning is related to the time and distance of the last encounter to the boundary. This phenomenon is indicating that the boundary cells are the neural bottom layer for error correction.

The boundary cells are located in the inferior hippocampus pad, which can make specific firing responses to the boundary of the environmental, and respond to different distances and angles. It plays a crucial role in space navigation and memory during mammals activating in wide-area environments. It is found that the rats cut the environment into multiple regions according to the space boundary during the navigation process. The expression of space by the grid cells is independent of the area enclosed by the boundary.

We extended the Continuous attractor network model of grid cells. The activation signal was input into the grid cells when the boundary cells were activated in the boundary region. Since the grid cells firing model was based on the stripe cells model, we only needed to extend the dynamics model of stripe cells.


(6) $$\begin{array}{l} \tau \frac{ds_{i}}{d_{t}}+s_{i}=f\left[\sum_{j}W_{ij}s_{j}+B_{i}+W_{id}B_{d}\right] \end{array}$$

The activation value of boundary cell *d* is *B*_*d*_, and the activation intensity of boundary cell is *c* for a given boundary region *R*_*d*_, so:


(7) $$W_{id}=\frac{1}{N}\int_{R_{d}}r_{i}(\vec{x})d\vec{x}$$

*N* represents the number of grid cells in the neural plate, and $r_{i}\left({\vec{x}}_{0}\right)$ represents the firing rate of grid cell *i* when the robot is at position ${\vec{x}}_{0}$. The weight of synapses from boundary cell *d* to grid cell *i* is proportional to the integral of grid cell firing in the *R*_*d*_ region:


(8) $$\begin{array}{l} W_{id}=\frac{1}{N}\underset{R_{d}}{\int }r_{i}\left(\overset{⇀}{x}\right)d\overset{⇀}{x} \end{array}$$

**Figure 6:**
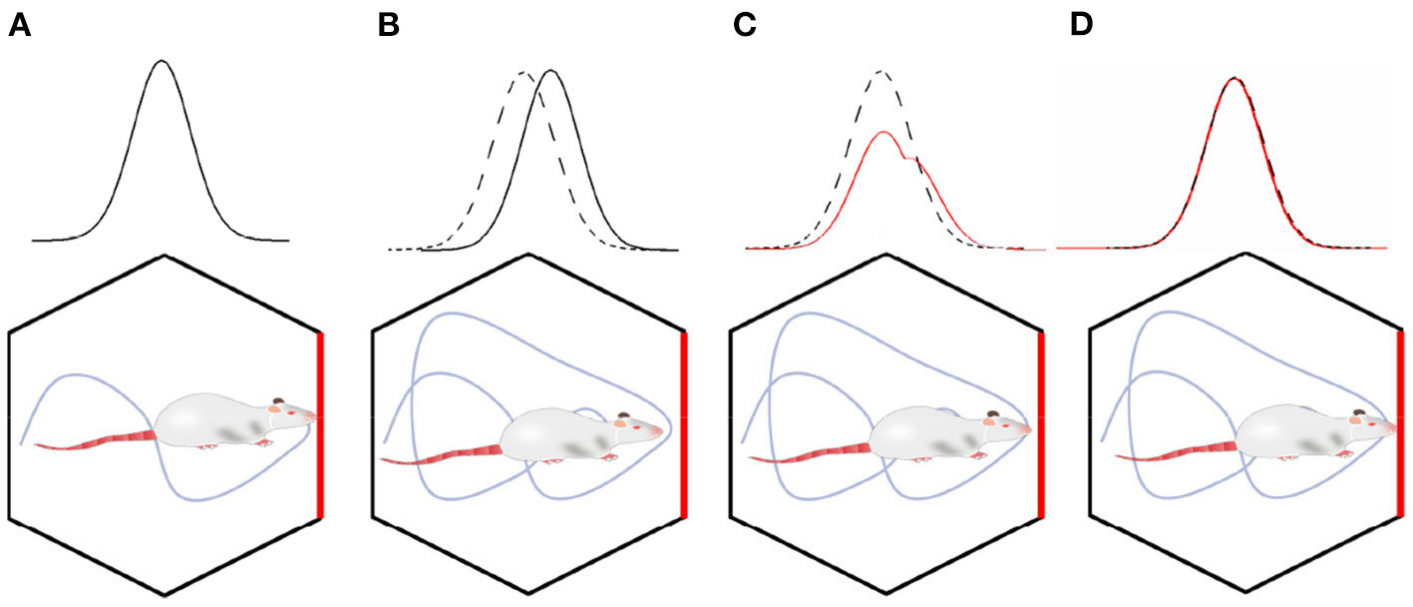
The mechanism of boundary cells correcting spatial cognitive errors. **(A)** In the initial exploration phase, the grid cells' activation pattern and boundary cells' activation pattern are associated with memory. **(B)** When the boundary cells are activated again, the grid firing pattern has deviated. **(C)** Boundary cells correct grid cell pattern by weight connection. **(D)** The error correction has been completed, the memory of grid cells activation pattern and boundary cells activation pattern are updated.

In the process of environment exploration for the robot, the boundary activation value of boundary cells was superimposed on the grid cells through the synaptic weight. It enhanced the intensity of the grid firing in the corresponding boundary region. Thus, grid cells realize the coding of the boundary information. In order to more intuitively explain the error correction mechanism by boundary cells, as shown in Figure 6, we simplified the error correction by a single boundary. As shown in Figure 6A, boundary cells are activated during the environmental exploration stage, and the activated information is remembered by the grid cells. As shown in Figure 6B, when the boundary cells are activated again, the grid firing pattern has deviated from the previous memory. As shown in Figure 6C, the remembered average activation value of the remembered boundary cells is weighted and projected onto the grid cells. Since the grid cells model is the superposition of stripe cells, according to the stripe cells dynamics function, the grid firing pattern will shift to the phase direction of the boundary cells weight matrix. As shown in Figure 6D, the final boundary cells realize the error correction for grid pattern through the memory of the weight matrix.

### Decoding Method of Spatial Information

After the grid cells complete the movement path's integration, it requires to decode spatial information to achieve expression on the place cells. Experimental and theoretical neuroscientists noticed the spatial periodicity firing of grid cells. They considered the grid cells as a metric of space (Hafting et al., 2005; Stemmler et al., 2015). The firing of grid cells is discretely spaced, so the phase of firing pattern can estimate the displacement of animal movement. Here we proposed a decoding method that was biologically feasible and could be calculated in real-time on a robotic system.

#### Simplification of Continuous Attractor Model

The continuous attractor net model is a bionic model that simulates the electrophysiological principle of nerve cells. The model realizes accurate path integration, and theoretically explains the phenomenon observed in biological experiments. However, It is difficult to use the existing mathematical methods to derive and calculate. So in the process of deriving the decoding algorithm, it is necessary to simplify the description of neural firing patterns through explicit mathematical functions. Therefore, we use explicit mathematical functions to replace the dynamics equation in the decoding operation. The Von Mises function is used to fit the firing characteristics of stripe cells Ω_*j*_(*x*) = *n*_max_ · *exp*{κ[cos(2π(*x* − *c*_*j*_)/λ) − 1]}. It is a periodic extension of the Gaussian function, where *n*_max_ is the maximum expected firing rate, *c*_*j*_ is the spatial priority phase of cell *j*, κ is the gain factor, and λ is its spatial period. The spatial firing probability function of the grid cells is the sum of three fringe waves rotated at an angle of 60 degrees. We model the grid cells as a function $\Omega_{j}\left(\vec{x}\right)$ that describes the average firing rate of the grid cells when the rat is in position $\vec{x}=\;\left(x,y\right)$:


(9) $$\begin{array}{l} \Omega \left(\overset{⇀}{x}\right)=n_{\max}\cdot \exp\left[\kappa /3\overset{3}{\underset{l=1}{\sum }}\left\{\cos\left(w{\overset{⇀}{k}}_{l}\cdot \overset{⇀}{x}\right)-1\right\}\right] \end{array}$$

Where the wave vector corresponding to each stripe cell is *k*_*l*_ = (cos(ϕ_*l*_), *sin*(ϕ_*l*_)), where ϕ_*l*_ = −π/6 + *l* · π/3.

#### Grid Cells Decoding Method

Each grid cell on the neural plate has the same scale λ, but different spatial phases. In order to decode the location of the robot by the firing activity of the grid cells population, we recorded the firing rate of grid cell *i* at the current location as *n*_*i*_. Thus, the response vector of the grid neural plate population is $\vec{n}=\left(n_{1},\ldots ,n_{n}\right)$. The average firing rate of neuron *j* is $\Omega_{j}\left(\vec{x}\right)$ when the robot in position $\vec{x}$ The real numbers ${\vec{n}}_{j}$ are scattered around this value. We assume that the firing rate $\Omega_{j}\left(\vec{x}-{\vec{c}}_{j}\right)$ of grid cell *j* obeys the Poisson distribution and that each neuron is statistically independent, so the probability of the firing rate vector at the position $\vec{x}$ of a given population composed of *M* cells is:


(10) $$\begin{array}{l} p\left(\overset{⇀}{x}|\overset{⇀}{n}\right)\propto \overset{M}{\underset{j=1}{\prod }}\Omega {\left(\overset{⇀}{x}-{\overset{⇀}{c}}_{j}\right)}^{n^{j}}/n_{j}!\cdot \exp\left(-\Omega \left(x-{\overset{⇀}{c}}_{j}\right)\right) \end{array}$$

Considering that the grid firing covers the movement space uniformly, so $\overset{M}{\underset{j=1}{\sum }}\Omega_{j}\left(x\right)$ is approximately a constant, the above joint probability density function of each grid cell can be simplified as:


(11) $$\begin{array}{l} P\left(\overset{⇀}{x}/\overset{⇀}{n}\right)=C\cdot \exp\left(\kappa /3\overset{M}{\underset{j=1}{\sum }}\overset{3}{\underset{l}{\sum }}n_{j}\cos\left(\omega {\overset{⇀}{k}}_{l}\cdot \left(\overset{⇀}{x}-{\overset{⇀}{c}}_{j}\right)\right)\right) \end{array}$$

The maximum likelihood estimate $\vec{x}={\vec{\mu }}^{ˆ}$ can be obtained from the above equation, where:


(12) $$\begin{array}{l} {\vec{\mu }}^{ˆ}=2/3\overset{3}{\underset{l=1}{\sum }}\mu_{l}{\vec{k}}_{l} \end{array}$$

(13) $$\begin{array}{l} \mu_{l}={\vec{k}}_{l}\cdot {\vec{\mu }}_{l}=\omega^{-1}\arg\left(\sum_{j=1}^{M}n_{j}\cdot \exp\left(i\omega {\vec{k}}_{l}\cdot {\vec{c}}_{j}\right)\right) \end{array}$$

#### Multi-scale Grid Cells Decoding Method

**Figure 7:**
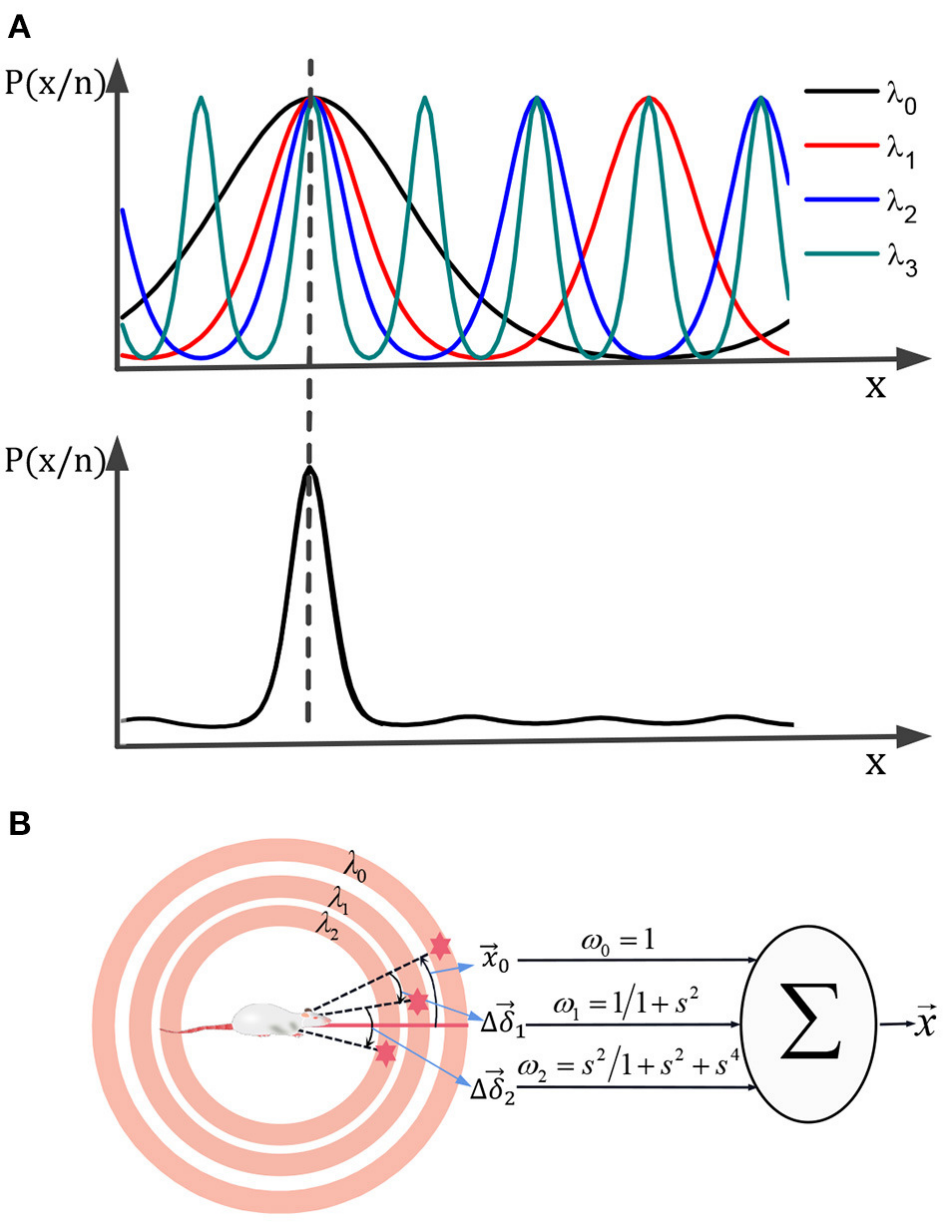
A unique high-precision solution can be obtained by calculating the joint distribution of the posterior probability of each scale grid neural plate **(A)**. **(B)** The multi-scale decoding could be calculated by the weighted sum of the deviations.

Suppose the scale of a single grid period is relatively large and can cover the range of motion. In that case, It can get a unique position solution according to the above decoding method, but the uncertainty error of the decoding will be relatively large. Using a smaller grid period scale can reduce the uncertainty error, but it will generate multiple solutions. Referencing nesting probability calculation method (Stemmler et al., 2015), multiple scaled-down grids' cell neural boards can be used for joint decoding. It could obtain a unique position solution with high precision. The principle is shown in Figure 7A. Combining various scale probability distributions of solutions in various scales, get the joint probability distribution. The maximum likelihood solution of the joint probability distribution is the final solution for the robot's actual location.

We assume that there are *m* grid neural plates with different scales. The number of nerve cells on each neural plate is *M*_*m*_, and the grid direction is the same. The maximum grid period is λ_0_, and the grid period of the *m* th grid neural plate is λ_0_/*s*_*m*_. So, the posterior probability is:


(14) $$\begin{array}{l} P\left(\overset{⇀}{x}/\overset{⇀}{n}\right)=C\prime \cdot \exp\left(\kappa /3\overset{M_{m}}{\underset{j=1}{\sum }}\overset{3}{\underset{l}{\sum }}n_{j}\cos\left(\omega s_{m}{\overset{⇀}{k}}_{l}\cdot \left(\overset{⇀}{x}-{\overset{⇀}{c}}_{j}\right)\right)\right) \end{array}$$

The maximum likelihood solution based on multi-scale joint posterior probability is:


(15) $$\begin{array}{l} x_{ML}=\overset{L-1}{\underset{m=0}{\sum }}M_{m}s_{m}^{2}{\overset{⇀}{\delta }}_{m}/\overset{L-1}{\underset{m=0}{\sum }}M_{m}s_{m}^{2} \end{array}$$

Where, ${\vec{\delta }}_{m}$ represents the position solution on the *m* th grid neural plate. Equation (15) can calculate the joint maximum likelihood solution of *L* grid cells neural plates. Set the number of cells of each neural plate to be equal, set $\Delta {\vec{\delta }}_{L}={\vec{\delta }}_{L}$*-*${\vec{x}}_{L}$, and *s*_*m*_ = λ_0_/λ_*m*_. From above, we set ${\vec{x}}_{L}$ as the maximum likelihood estimate by the combination of *L* neural plates. Then ${\vec{x}}_{L+1}$ can be calculated by the following recursive formula :


(16) $$\begin{array}{l} {\vec{x}}_{L+1}={\vec{x}}_{L}+{\lambda_{L}}^{-2}/\left(\overset{L}{\underset{m=0}{\sum }}\lambda_{m}^{-2}\Delta \delta_{L}\right) \end{array}$$

According to Equation (16), the decoding from grid cells to place cells can be iterated according to the following steps:

(1) Start the decoding calculation from the scale λ_0_ that a cycle can cover the movement space, and calculate the population vector activation value to obtain the roughest displacement estimate ${\vec{x}}_{0}$.
(2) Using the estimated value ${\vec{x}}_{0}$ as the center, calculate the relative offset value of position estimation on the grid cells neural plate of the scale λ_1_, and multiply the offset value by the weight value to correct the estimated value of the previous scale, to obtain a new displacement estimation value ${\vec{x}}_{1}$.
(3) Similar to the previous step, new displacement estimates ${\vec{x}}_{2}$, ${\vec{x}}_{3}\cdots \;$ are calculated step by step.

As shown in Figure 7B, the multi-scale decoding calculation process can be regarded as the weighted summation process of deviation values, which can be realized physiologically by calculating neural synapses.

### Bionic Spatial Cognition Model

**Figure 8:**
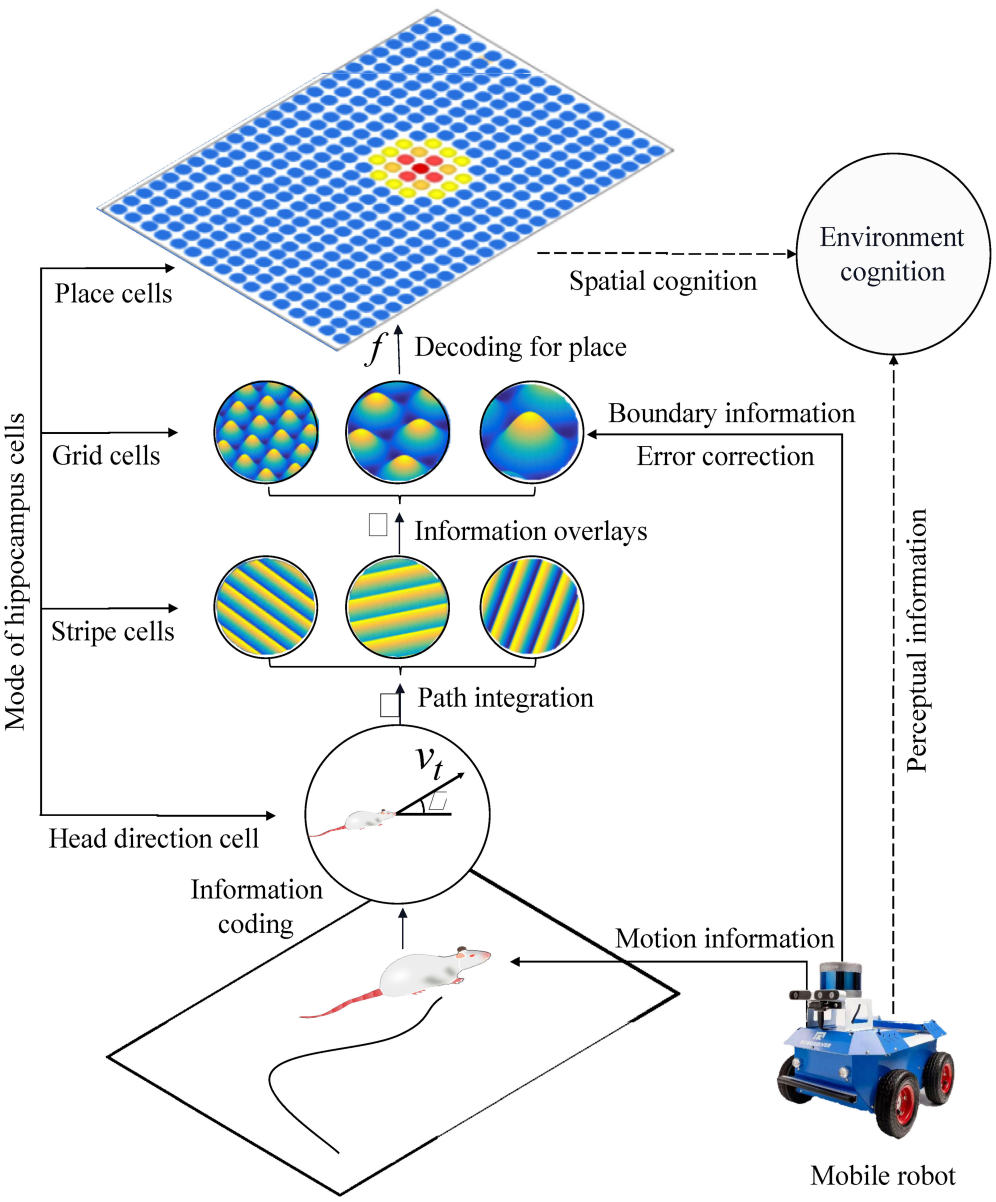
The figure shows the system architecture of the bionic spatial cognitive model on the mobile robot. The process of robot spatial cognition includes the following steps: (1) Head direction cells and stripe cells code the robot's motion information and integrate the paths. (2) The neural information from stripe cells to grid cells is integrated into two-dimensional space. (3) The boundary information corrects the grid cells integration error according to the memory of synaptic weight. (4) By decoding the firing signals of multi-scale grid cells, our model realized the one-to-one expression of physical space.

Based on the models of spatial cells in the hippocampus: head direction cells, stripe cells, grid cells, place cells, and boundary cells, we constructed a bionic spatial cognitive model for robots. As shown in Figure 8, the system consisted of a mobile robot chassis, a compass sensor, an ultrasonic sensor, and a notebook computer. The front wheels of the mobile chassis are equipped with two incremental encoders that provide raw motion data, a compass that provides directional information, and ultrasonic sensors are mounted around the mobile chassis to provide environmental boundary information. The movement velocity and direction of the robot are encoded in the firing activities of the head direction cells, and the boundary information is encoded in the firing activities of the grid cells. The calculation process includes four main steps:

(1) The robot's motion information is encoded by head direction cells, then stripe cells realize one-dimensional spatial cognition through path integration.
(2) The spatial cognition is extended to a two-dimensional spatial expression of grid cells by neural projection from stripe cells to grid cells in multiple priority directions.
(3) The boundary information is encoded into the firing activity of the grid cells. When the boundary signal is detected again, the grid cells will self-correct the accumulated error according to the memory of the synaptic weight.
(4) By decoding the firing signal at the multi-scale grid cells, the precise position information of the place cells could be obtained, and the one-to-one expression of the physical space is realized.

## Experiment and Result Analysis

### Spatial Cells Path Integral Experiment and Results

#### Results of Stripe Cells Experiment

**Figure 9:**
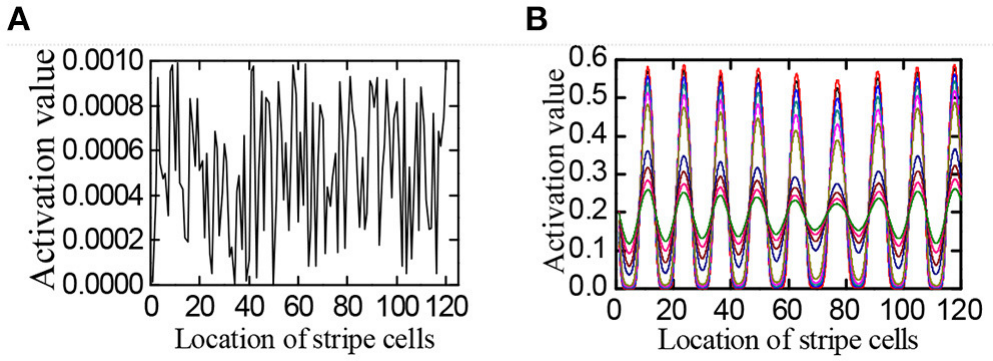
The process of stripe formation. **(A)** The initial firing of the stripe cells neural plate under the action of noise. **(B)** Striped firing pattern were formed spontaneously over time, the curve from green to red, respectively, represents the striped firing pattern from 50 to 500 *ms*.

**Stripe formation:** There are 120 stripe cells arranged in the longitudinal direction of the stripe cells' neural plate. In the beginning, the robot is stationary, that is, the actual velocity $\vec{\nu }=$ 0. Due to the influence of sensor noise, the input value to the head direction cells is a slight value noise. Upon that, the stripe cells spontaneously form a stripe firing pattern. As shown in Figure 9, the formation process of the stripe firing pattern is within 500*ms*.

**Stripe movement:** Based on the firing pattern of the stripe cells formed, the robot roamed in a 1*m* × 1*m* environment and projected the speed information into the stripe cells model to drive the stripe wave moving. We had calculated the correspondence between the displacement of the robot and the shift of stripe wave. Experiments had proved that the robot's motion could produce highly linearly correlated stripe movement.

**Figure 10:**
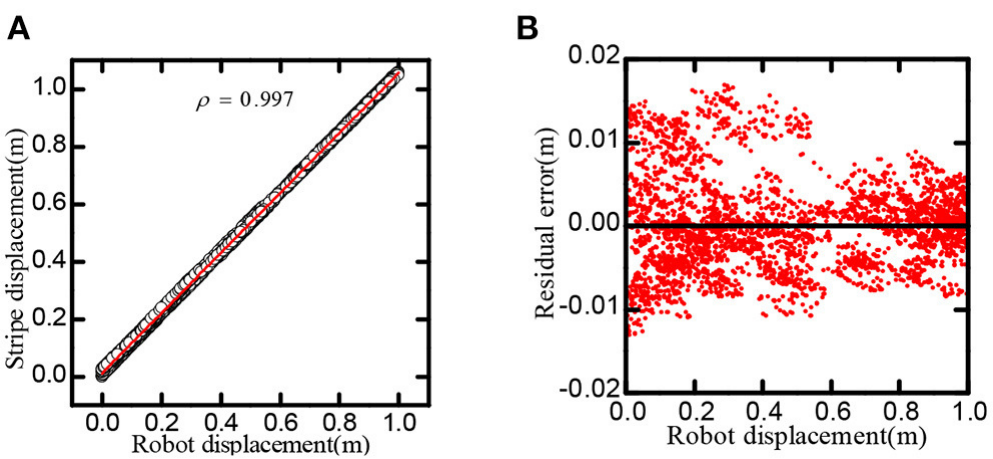
Stripe-driven experimental result. **(A)** The linear relationship between the position and the phase movement of the stripe. **(B)** The direct residual of the stripe cells on cognitive position and regression line.

As shown in Figure 10, the linear correlation reaches 0.9997, in one-dimensional space, the residual of the regression line is less than 0.02*m*.

**Figure 11:**
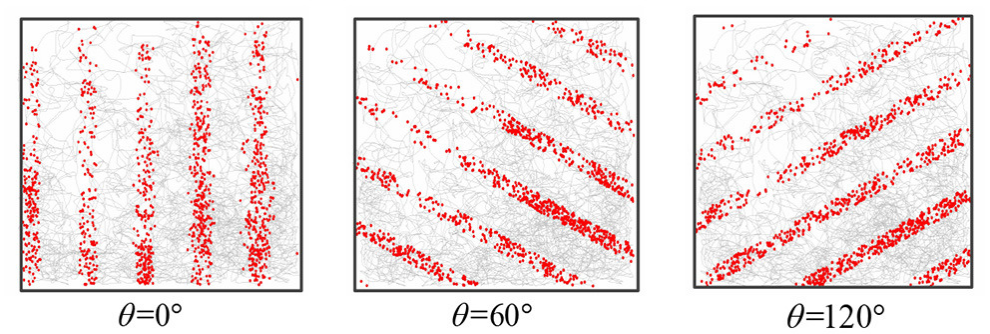
The figure shows the firing point of a single stripe cell in the trajectory. Striped cells neural plates with different priority directions can form patterns in different directions.

**One-dimensional path integral:** When adjusting the direction of the synaptic connection, the stripe cell neural plate could obtain striped firing fields in different orientations. The robot movement time was *t* = 5, 000 *s*, the stripe spacing was *L* = 0.2*m*, and the stripe direction θ is 0, 60, and 120 degrees, respectively. Figure 11 recorded the firing rate of the single-cell in-motion trajectory, which shows that the firing field of stripe cells can form an accurate displacement integral in the specific direction.

#### Results of Grid Cells Experiment

**Figure 12:**
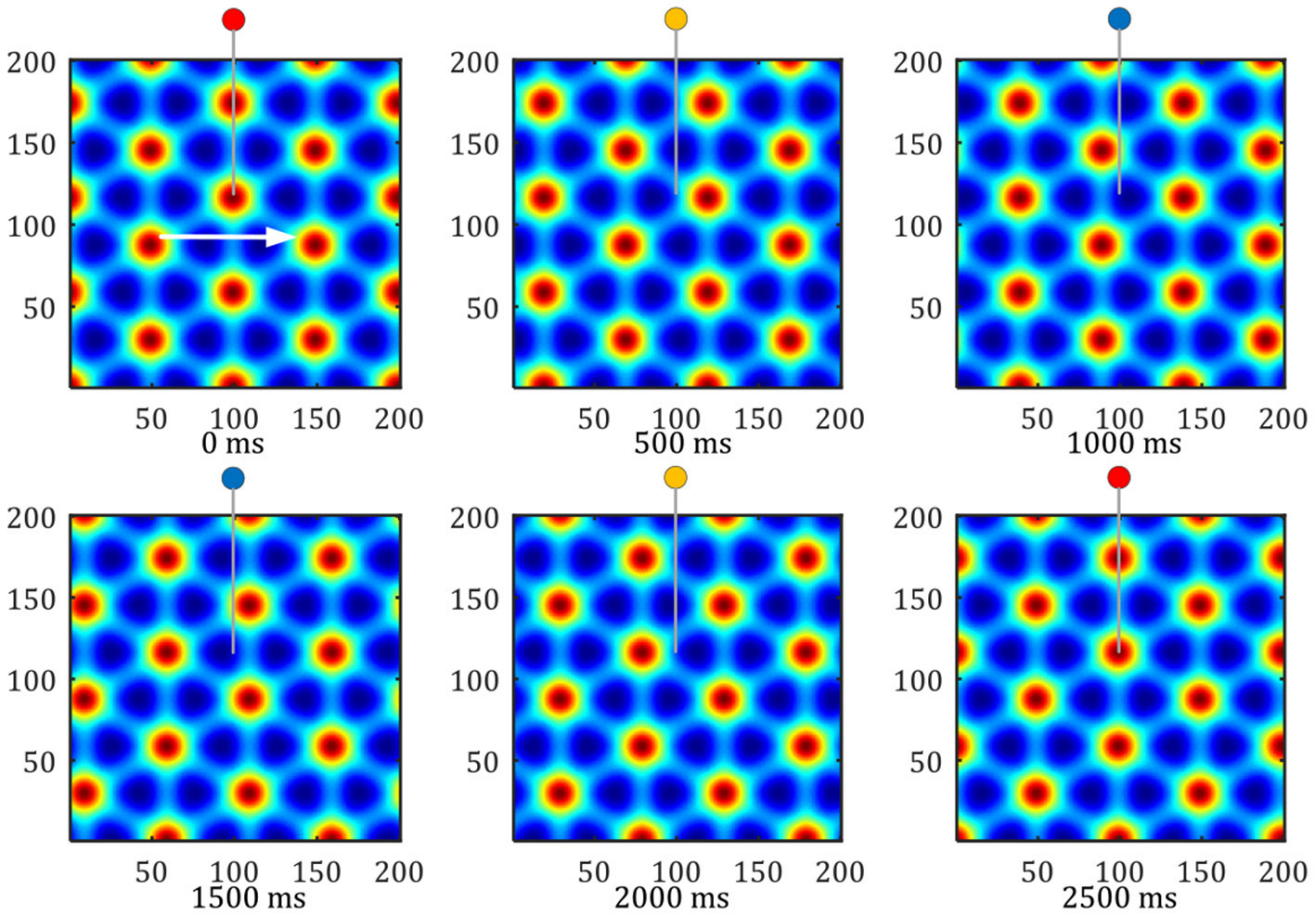
Pattern movement of grid firing. The red circle represents the peak firing period of a single grid cell, and the blue represents the low firing period.

**Grid formation:** Set the number of neurons in the grid cells neural plate as 200 × 200, set the stripe cells' priority direction to 0, 60, 120 degrees. The activation values of the stripe cells are superimposed and projected onto the grid cells neural plate, to form a grid-like firing pattern. As shown in Figure 12, The hexagonal grid formed can cover the entire space of robot movement. The input of the stripe cells of different intervals can obtain the grid patterns of different intervals.

**Grid movement:** The grid movement can be measured from two methods. One method is to sample the activation values of the grid cells population on the entire neural plate at different times to compare the phase changes of the grid mode. The other is to record the periodic firings of a single grid cell, Which indirectly reflects the moving distance of the grid discharge pattern. As shown in Figure 12, the white arrow shows the direction of the robot's uniform motion. The activity pattern on the neural plate of the grid cells flowed along the direction of movement, and one grid period movement was realized within 2, 500 *ms*. In this process, the single grid cell on the neural plate realized a periodic firing. The red circle represents the peak firing period of a single grid cell, and the blue represents the low firing period.

### Simulation and Results of Boundary Cells Correcting Accumulation Error

**Figure 13:**
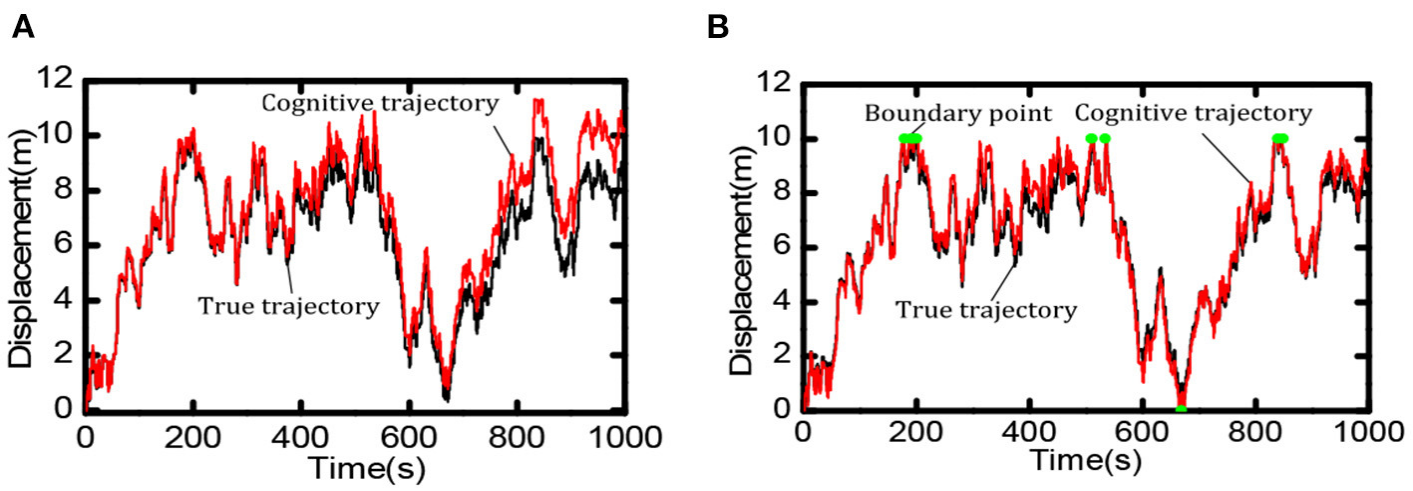
Comparison of spatial cognition accuracy between with and without boundary cells. **(A)** When there is no boundary cell, the deviation between the cognitive results and the actual trajectory becomes larger and larger. **(B)** When touching the boundary (at the green point) the accumulated error is corrected.

Boundary information can effectively reduce the accumulated errors of the grid cells and provide an essential foundation for the robot's long-term and stable spatial cognition. We compared the robot's cognition trajectory without and with the boundary cells within 0 ~ 1,000 *s*. As shown in Figure 13A, when there was no boundary cell, due to the accumulation of errors the cognitive results of the robot in space deviated more and more from the actual trajectory. When there were boundary cells, as shown in Figure 13B, when the robot touches the boundary (the red dot in the figure indicates), the cognitive trajectory was corrected due to the weight effect of boundary cells, the accumulated errors did not accumulate continuously.

### Spatial Position Decoding of Multi-Scale Grid Cells

**Figure 14:**
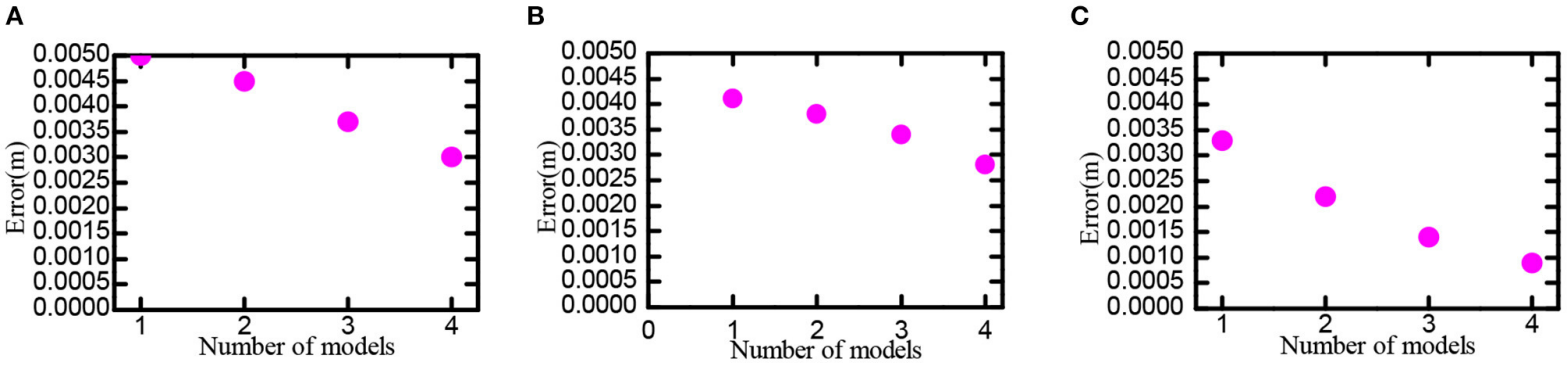
Position decoding experiment for grid cells. Part labels **(A–C)** are the experimental data when the number of cells in the grid neural plate is 50 × 50, 100 × 100, and 200 × 200.

Set scale ratio between the neighboring neural plate as *i* = 1.5, feed the information of neural plates in various scales into the decoding model, try to use 1, 2, 3, and 4 scales, respectively decoding the space position. The neural plate in tbe experiment was constructed with 50 × 50, 100 × 100, and 200 × 200 grid cells, respectively. Experimental results are shown in Figure 14. In the case of the same number of grid cells, the larger the number of multiscale neural plate combined decoding could significantly reduce the decoding error. Increasing the number of nerve cells could significantly reduce the decoding error.

### Experimental Results of Spatial Cognition of Mobile Robots

**Figure 15:**
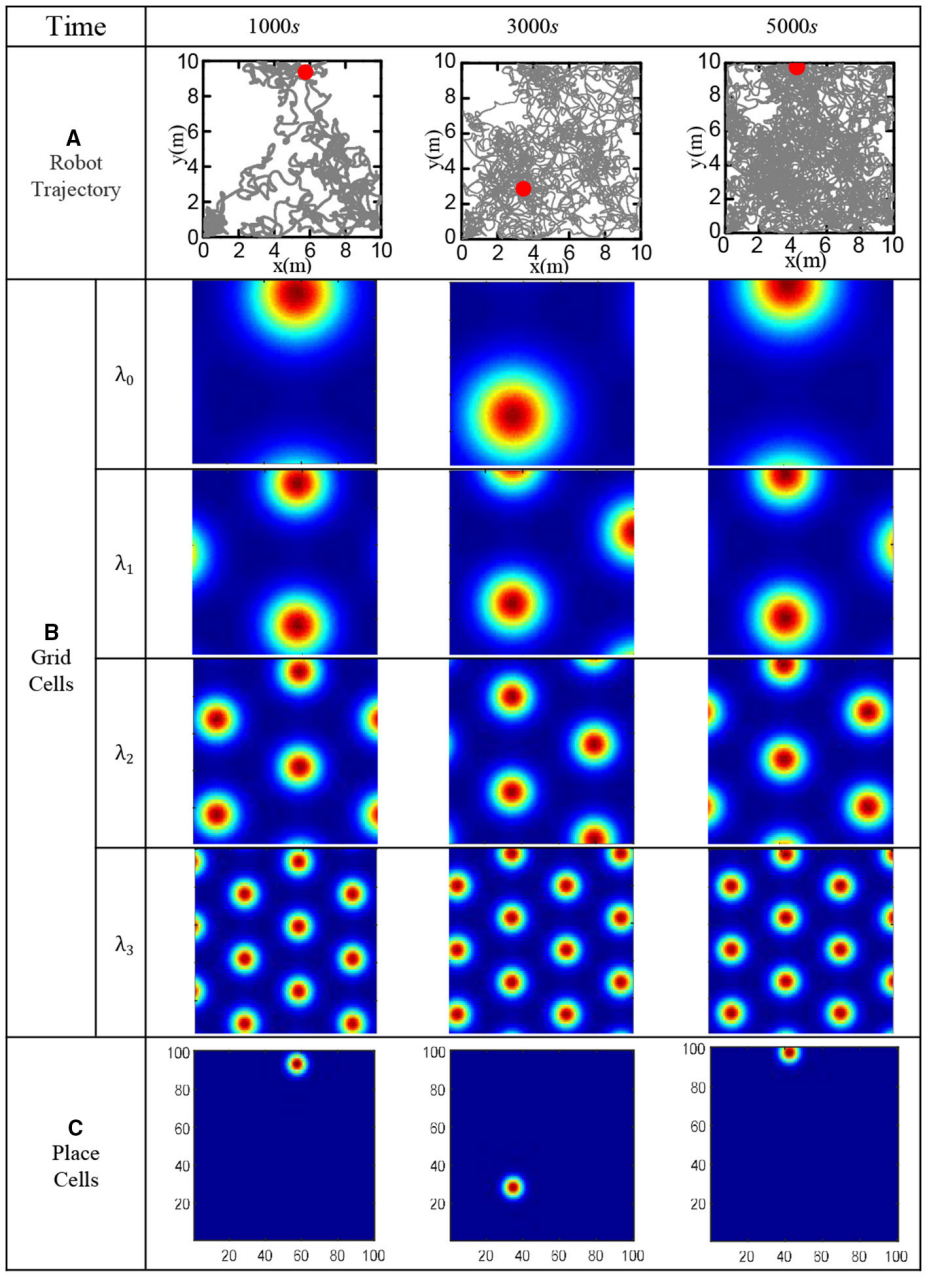
Functional verification of robot bionic cognitive model. **(A)** The gray line is the robot's trajectory, and the red dot is the robot's position at the current time. **(B)** The population firing pattern of grid cell neural plate at each time and each scale. **(C)** Firing pattern of place cells at each time.

In order to verify the effectiveness of the bionic spatial cognition model, the robot walked randomly in the boundary area of 10 × 10 *m*^2^ for 5, 000 *s*. Set the movement speed to a random number in (0, 0.5) m/s, and the direction to a random number in (−π/2, π/2). Use the robot to imitate the rat's irregular free exploration movement in the room. The specific speed and direction information of the robot can be obtained from the gyroscope and the encoder, then fed into the model. The roughest grid period is set at λ_0_ = 10*m*, and the grid-scale ratio of all levels is *i* = 1.5. Figure 15A shows the actual trajectory positions of the robot at various moments. Figure 15B shows the population firing patterns of the grid cells at each scale at different moments, which represents the integral results of the grid cells to the spatial path. Figure 15C shows the place cells' firing response after decoding the grid cells at each moment. The obtained cognitive expression of place cells corresponds to the actual trajectory of the robot, which proved that the bionic spatial cognition model we constructed could realize the cognition of the environment and form a firing response corresponding to the location of the space.

**Figure 16:**
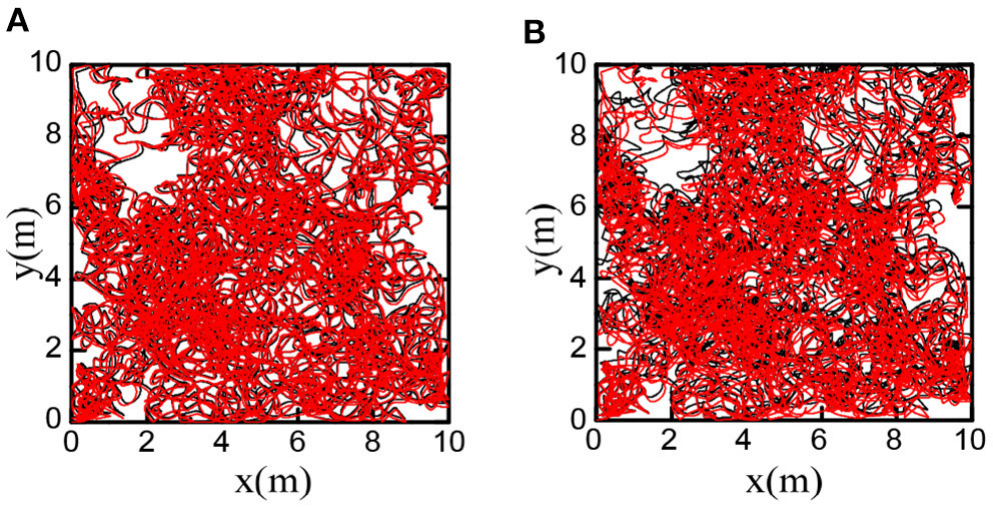
Comparison of bionic cognition and odometer trajectory error, the black curve represents the real trajectory of the robot. **(A)** The red curve represents the bionic cognitive trajectory. **(B)** The red curve represents the odometer trajectory. Obviously, the bionic spatial cognitive trajectory is closer to the real trajectory.

We decoded firing patterns of grid cells at each time step to obtain the cognitive trajectories, which were used as the spatial cognition map of the robot. Figure 16A shows that the bionic spatial cognitive trajectories kept a slight deviation from the actual trajectories. For comparison, we accumulated the robot encoder to obtain the odometer trajectory. Figure 16B shows the odometer trajectories with significant deviation for the accumulated errors after a long time of movement.

**Figure 17:**
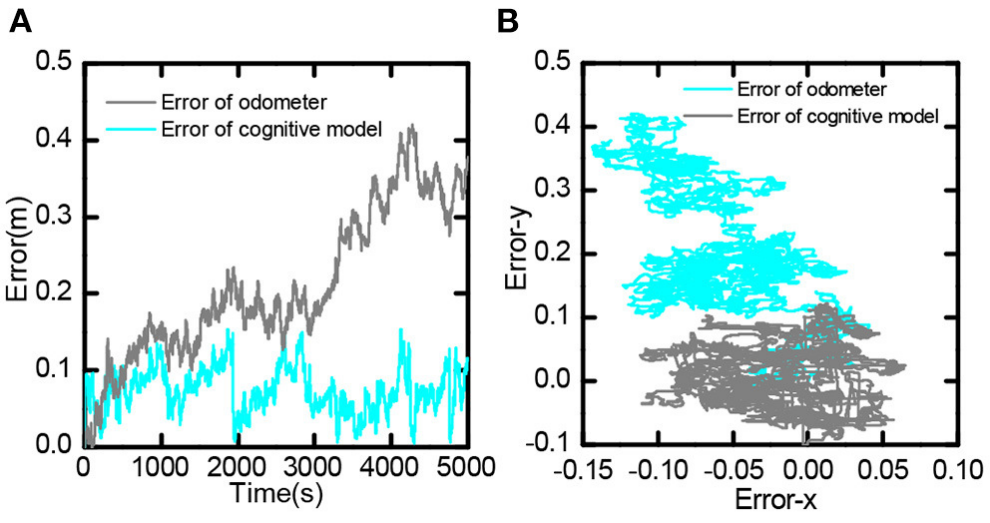
Bionic spatial cognitive model and odometer error comparison, **(A)** Comparison of error value from 0 to 5,000 s. **(B)** Comparison of the distribution of error values in the entire trajectory.

It compared the error value between the bionic spatial cognition and the odometer based on the encoder within 0-5, 000 *s*. As shown in Figure 17A, the position error obtained by the odometer continued to expand. In contrast, the position error based on the bionic cognitive model was always stable within a small range. Figure 17B shows the distribution of errors in the process of movement. The position error of the odometer was biased to one side of the point (0,0), while the position error of the bionic cognition model was only around the point (0,0). This experiment proves that the bionic spatial cognitive model based on the proposed hippocampus has good cognitive accuracy and robustness to information error.

## Discussion

### Feasibility of Robot Application

The spatial cognition model proposed in this paper aims to stimulate the cognition mechanism of the hippocampus, which can be embedded into mobile robots to realize environmental cognition and autonomous navigation as humans and animals, explaining its feasibility from the following two points.

**Physiological feasibility:** (1) The constructed hippocampal cell models such as head direction, striped, grid, and boundary cells can simulate physiological firing responses as found in biological experiments. (2) The mathematical calculations in our algorithms, such as weighted summation, threshold value, exponential operation, and differential operation, can all be realized through the principles of neural synapses and membrane potentials.

**Robotics feasibility:** (1) The input velocity, direction, boundary, and other perception information required by our algorithm can be easily obtained on ordinary mobile robots. (2) We use population firings of neural cells to express the results of cognition calculations. The results are easily converted into electronic information for information transmission and processing.

### The Accuracy of Spatial Cognition

Humans and mammals can roughly recognize spatial position through their motion information. Then they can correct spatial cognition information to achieve precise positioning with the assistance of other perceptual information. For mobile robots, the input velocity is from the wheel encoder, and there is inevitably the influence of wheel slip, deformation, and sensing accuracy. The robot bionic cognition model we proposed uses the boundary signal captured by the distance sensor to correct the cognitive position, which could effectively eliminate accumulated errors and generate an accurate representation of the environment. With the deepening of research, integrating more perceptual information such as exogenous landmark information can further improve the accuracy of spatial cognition.

### The Advantages of the Proposed Method

**Figure 18:**
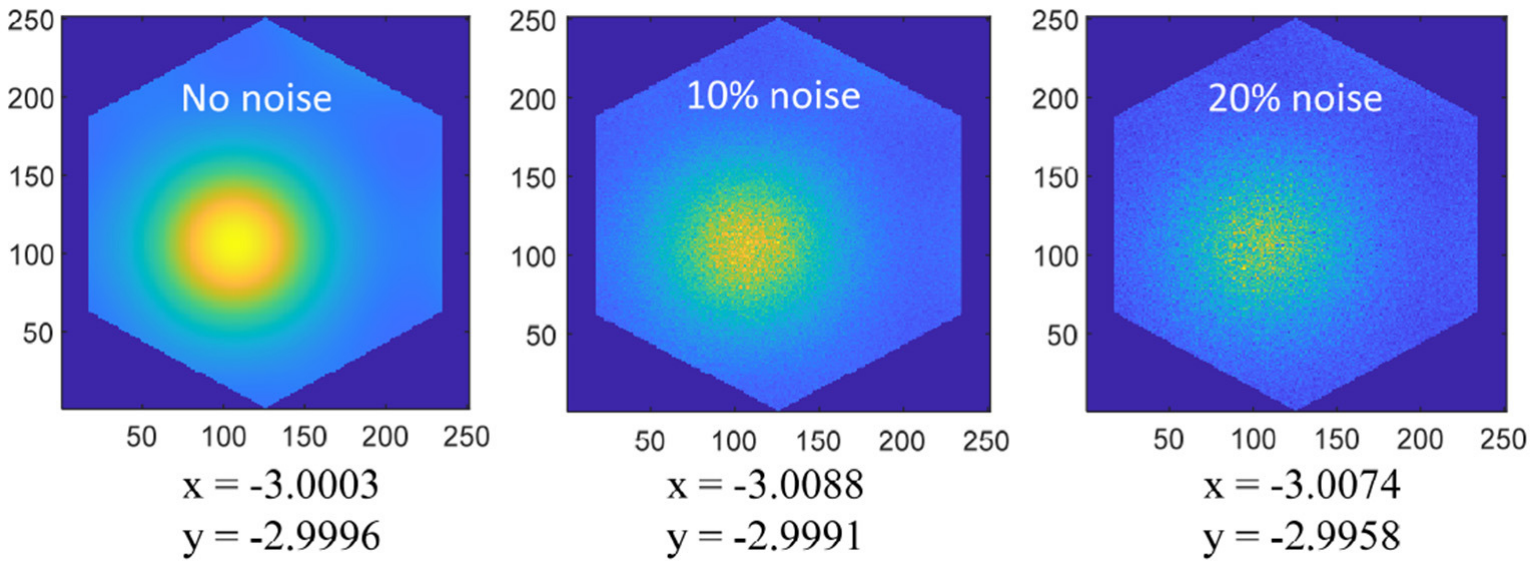
Coordinate positions (−3, −3) are coded on the 250 × 250 grid cell neural plate, and a single period of hexagonal lattice is intercepted according to the size of the grid period. Add 0, 10%, 20% amplitude Gaussian noise to the grid activation value, and then decode the space coordinates as shown in the figure, the noise has very little interference on the position expression.

The spatial cognition model and method we put forward mainly realize the neural expression of position information and the integration of the path. The realization of this part is to provide the basis for the subsequent research on the neural-inspired robot situational recognition algorithm. In the traditional SLAM algorithm, the spatial information is expressed by the symbolic value in the Cartesian coordinate system, and the integral of the path is the accumulation of the symbolic value. The method proposed in this paper has the following advantages: (1) The spatial information based on neural expression has a stronger anti-interference ability. As shown in Figure 18, Gaussian noises are added to the grid cells, then the position error is very little disturbed; (2) The spatial cognition model we proposed can achieve error correction through boundary information, and the sensor (ultrasonic rangefinder) used is cheaper than methods based on vision or laser; (3) Spatial expression based on grid cells is proved to be more suitable for the learning of artificial neural networks (Banino et al., 2018); (4) In the research field of bionic topology and vector navigation, the neural expression of grid cells and position cells is the basis of path planning and navigation. Compared with the navigation method of tradition, bionic topology and vector navigation are more suitable for wide-area, dynamic, and complex environments (Erdem and Hasselmo, 2012; Edvardsen et al., 2020).

### Current Deficiencies and Follow-Up Research

The experimental environment preset in this research is a small space area with a boundary. However, in the actual environment of mammals or mobile robots, the space area may be much larger or even without obvious environmental boundaries. When the space exceeds the maximum grid period, there is more than one decoded value from grid cells to place cells. The low-frequency path correction by the boundary cell will make the cumulative error greater and greater. So it is impossible to obtain accurate spatial cognition for the scenes which are too large or without boundaries. It is necessary to obtain a higher level of environmental cognition by combining more external perception information, such as vision, hearing, magnetic field, and other information, which will be solved in subsequent research.

## Conclusion

The three main contributions of this paper: (1) Based on the continuous attractor net theory, the working mechanism of the hippocampus was simulated and a series of spatial cell models was built, realizing the integration of the spatial information of the environment. (2) The memory capacity of boundary cells was imitated to realize the correction of spatial cognition information. (3) A set of methods was proposed for decoding and expressing the place information of grid cells to realize the one-to-one correspondence between the place cells and the physical space. In summary, the spatial cognition model and method based on hippocampus proposed in this paper are significant to intelligent robot navigation, environmental cognition, and map construction.

## Data Availability Statement

The original contributions presented in the study are included in the article/supplementary material, further inquiries can be directed to the corresponding authors.

## Author Contributions

JY made substantial contributions to the original ideas, designed the experiments, and wrote the manuscript. WG developed the simulation platform and performed the experiments. FZ typeset the manuscript and is accountable for the publishing issues. LS and ML provide financial support for the study. PW supervised, analyzed the results, provided feedback, and revised the manuscript. All authors contributed to the article and approved the submitted version.

## Funding

This work is supported by National Natural Science Foundation of China (NSFC) 61773139, National Natural Science Foundation of China (NSFC) 51521003, National Natural Science Foundation of China (NSFC) 52075115, Shenzhen Science and Technology Research and Development Foundation JCYJ20190813171009236, Shenzhen Science and Technology Program KQTD2016112515134654, National Natural Science Foundation of China (NSFC) U2013602, Self-Planned Task (NO.SKLRS202001B and SKLRS202110B) of State Key Laboratory of Robotics and System (HIT) and National Natural Science Foundation of China (NSFC) 61911530250.

## Conflict of Interest

The authors declare that the research was conducted in the absence of any commercial or financial relationships that could be construed as a potential conflict of interest.

## Publisher's Note

All claims expressed in this article are solely those of the authors and do not necessarily represent those of their affiliated organizations, or those of the publisher, the editors and the reviewers. Any product that may be evaluated in this article, or claim that may be made by its manufacturer, is not guaranteed or endorsed by the publisher.
